# Aggressive behavior of Her-2 positive colloid breast carcinoma: A case report in a metastatic breast cancer

**DOI:** 10.1016/j.amsu.2020.02.010

**Published:** 2020-03-10

**Authors:** Sumadi Lukman Anwar, Ery Kus Dwianingsih, Widya Surya Avanti, Lina Choridah, Suwardjo, Teguh Aryandono

**Affiliations:** a Division of Surgical Oncology, Department of Surgery, Dr Sardjito Hospital, Universitas Gadjah Mada, Yogyakarta, 55281, Indonesia; b Department of Anatomical Pathology, Dr Sardjito Hospital, Public Health, and Nursing, Universitas Gadjah Mada, Yogyakarta, 55281, Indonesia; c Department of Radiology, Dr Sardjito Hospital, Public Health, and Nursing, Universitas Gadjah Mada, Yogyakarta, 55281, Indonesia

**Keywords:** Colloid, Mucinous, Her-2 postive, Delayed diagnosis, ER, estrogen receptor, PR, progesterone receptor, HER2, human epidermal growth factor receptor 2

## Abstract

**Introduction:**

Colloid breast carcinoma is a rare form of invasive ductal cancer characterized by large amount of mucous deposition. It is considered as an indolent cancer that usually affects older women. Colloid breast carcinoma generally expresses estrogen and progesterone receptors but negative for Her-2. Recommended surgery and adjuvant treatment of colloid breast carcinoma is not well-established.

**Presented case:**

A 46 years-old woman presented as an aggressive colloid breast carcinoma showing skin ulceration, enlargement of multiple axillary lymph nodes and a metastasis in the pleura at diagnosis. The primary tumor showed strong positive expression of estrogen, progesterone as well as Her-2 receptors. The patient was treated with 6 cycles of paclitaxel and carboplatin followed by mastectomy, radiotherapy, and hormonal therapy. Patient tolerated the treatment course and showed improvement both in the locoregional control and pleural metastasis.

**Discussion:**

Colloid breast carcinoma with aggressive clinical course is rarely found. Nodal involvement as a sign of poor prognosis in colloid breast carcinoma ranges only between 12 and 19%. Therefore, axillary node clearance is usually excluded during the surgery of colloid breast carcinomas. However, in the presence of high-risk characteristics, mastectomy involving axillary lymph node dissection is still contentious. In patients with Her-2 overexpression, treatment using anti-Her2 (trastuzumab) is also still disputed in colloid breast carcinoma because of the higher resistance rates.

**Conclusion:**

Although clinically aggressive colloid breast carcinoma is rare, thorough clinical assessment and immediate treatment initiation will be beneficial for patients with high risk of relapse and metastatic spread.

## Highlights

• Colloid breast carcinoma with Her-2 overexpression, lymph node infiltration, and distant spread is rare.
• Recommended surgery for colloid breast carcinoma is still debatable.
• In elderly patients with colloid breast carcinoma, less radical surgery without axillary node clearance is preferred.
• Mastectomy with node dissection might be beneficial in high risk colloid breast carcinoma patients.

## 1 Introduction

Colloid breast carcinoma is a variant of ductal carcinoma typically showing nests of cells surrounded by large fragments of mucin and is therefore known as mucinous breast cancer [1,2]. It accounts for 2% of all breast carcinomas and is associated with low grade, low rates of local and distant metastases, and favorable prognosis [[1], [2], [3]]. In addition, it is less likely to infiltrate the axillary lymph nodes and is more responsive to treatment [2]. Transcriptomic study has revealed that colloid breast carcinoma is included in the luminal A molecular subtype [4]. Genomic study has also revealed the relatively low genomic instability and rare recurrent oncogene amplifications [4] underlying its tendency as a slowly growing tumor. Immunostaining of ER and PR is usually positive and Her-2 expression analyzed both with immunohistochemistry and fluorescence in situ hybridization is predominantly negative [4]. Study reported clinical perspectives and prognosis of locally advanced and metastatic Her-2 positive colloid breast carcinoma is still limited. We presented a rare case of Her-2 positive colloid breast carcinoma in a relatively young woman showing skin ulceration, positive axillary lymph nodes, and pleural metastasis at diagnosis and reported the case in accordance to the SCARE 2018 guidelines [5].

## 2 Case report

**Fig. 1:**
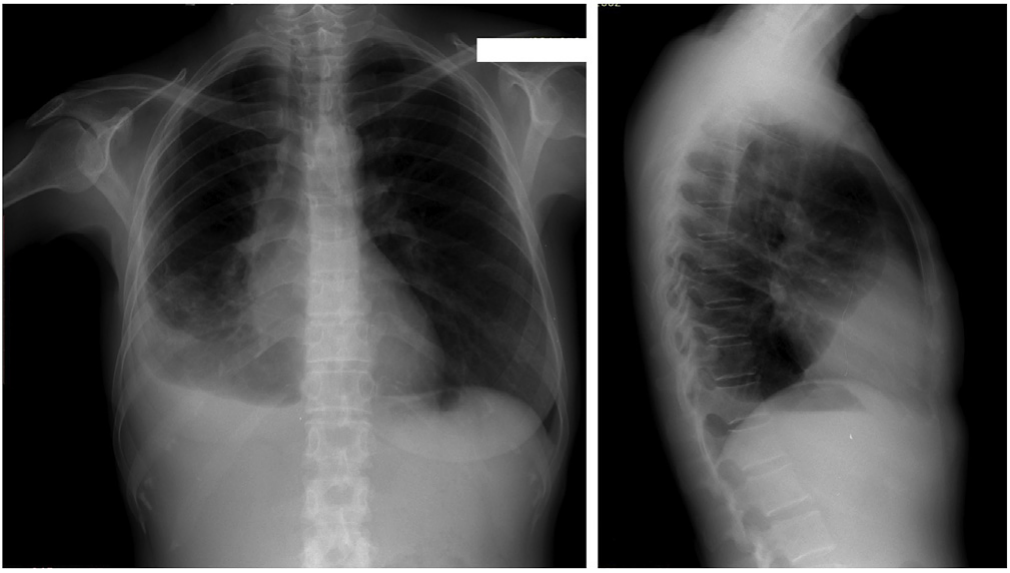
Chest X-ray at the emergency room showed a moderate right pleural effusion. In a circumstance with suspected malignancy as shown in this patient with lump and skin ulceration of the right breast with short of breath, a unilateral pleural effusion should be considered as metastatic disease. Cytology of pleural fluid confirmed the presence of malignant ductal cells.

**Fig. 2:**
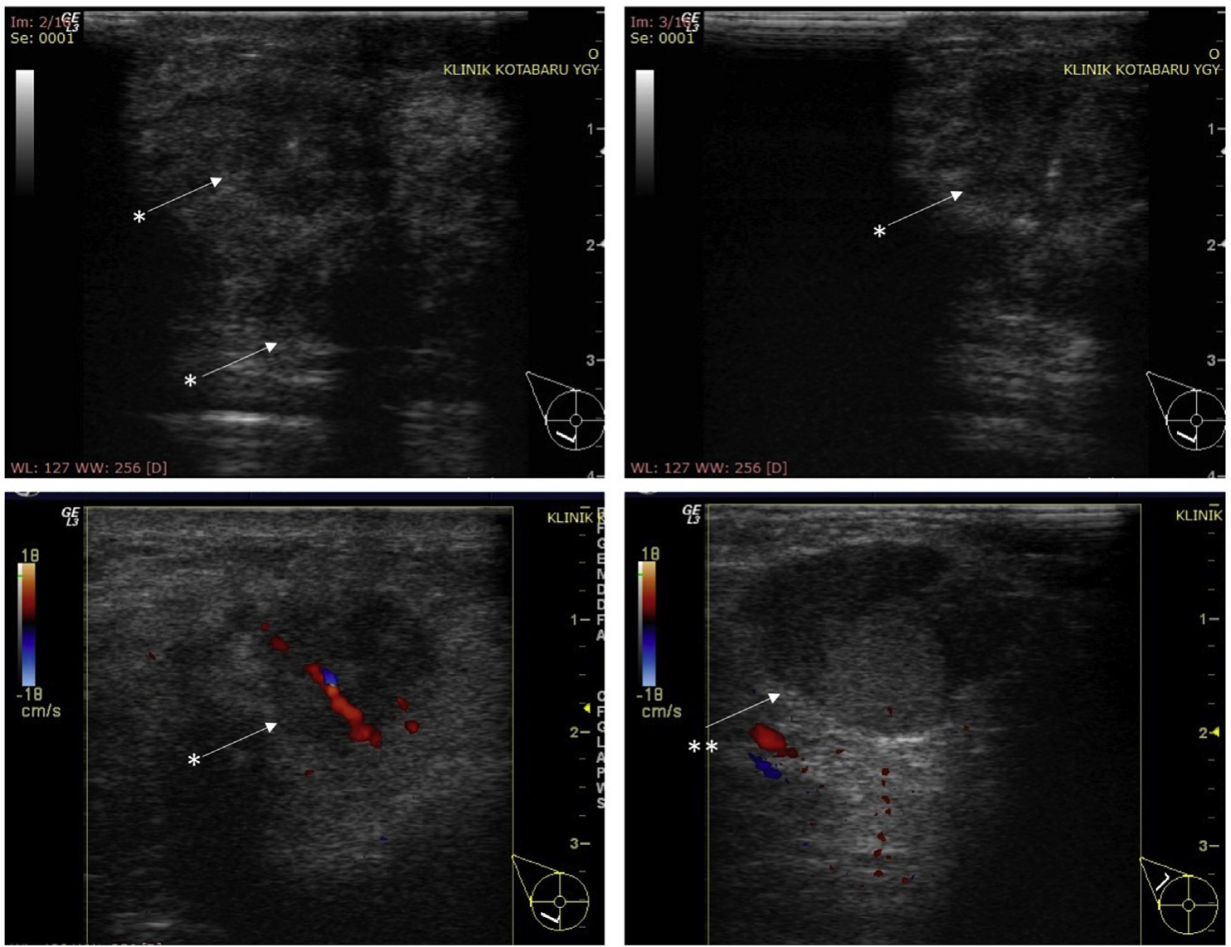
Ultrasonography of the right breast revealed diffuse hypo-echoid masses (*) with irregular border as well as enlargement of multiple lymph nodes in the right axillae (**).

**Fig. 3:**
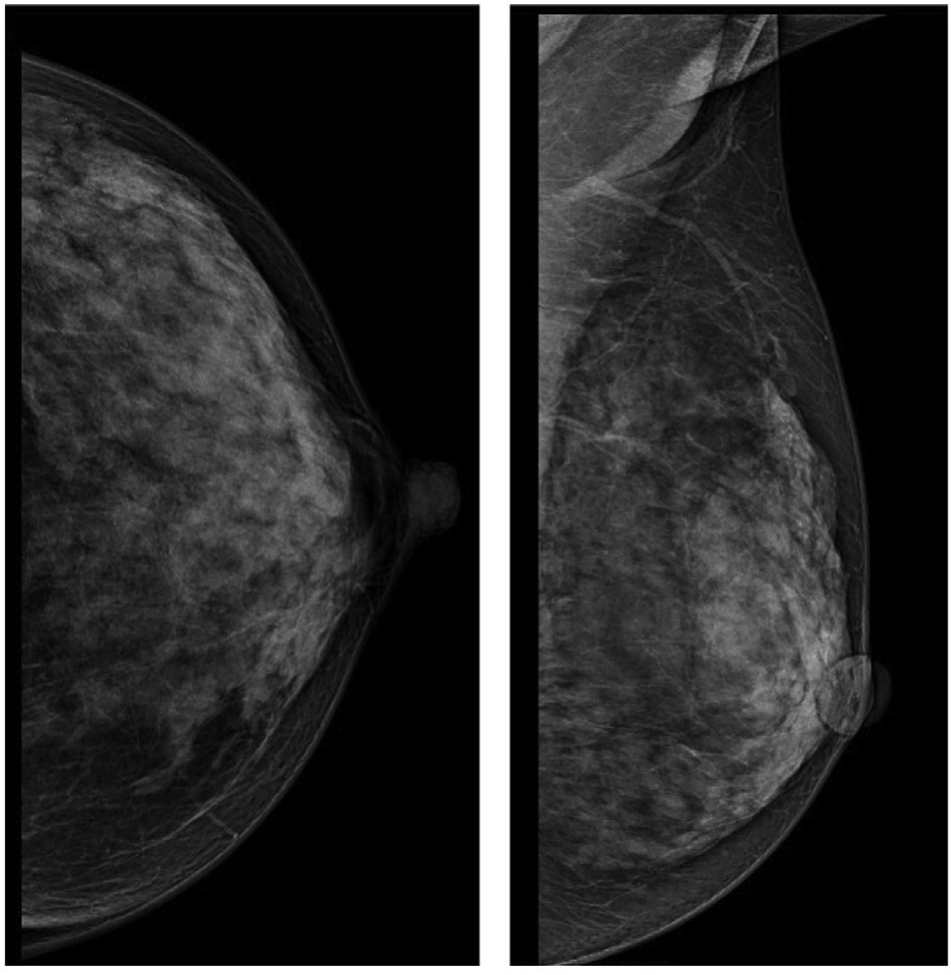
Mammograms of the affected breast could not be performed because of the large skin ulceration. Mammograms of the contralateral breast showed density class of D with an equal-density lesion located at the retro-papillae indicating a benign lesion.

**Fig. 4:**
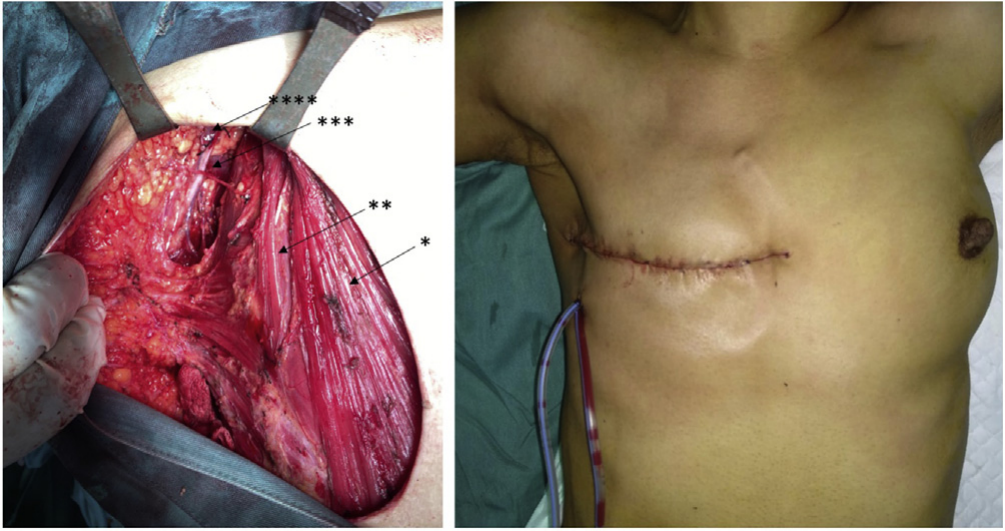
Modified radical mastectomy was performed after systemic chemotherapy resulted in the resolution of the pleural effusion exposing pectoralis major muscle (*), pectoralis minor muscle (**), thoracodorsal bundle (***), and axillary vein (****).

**Fig. 5:**
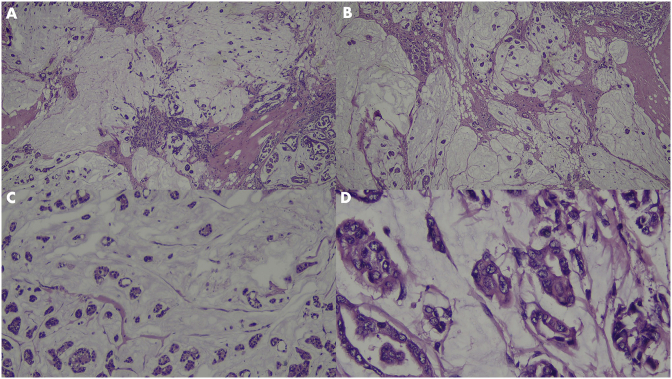
Panoramic view of histopathological features showed a large portion of mucin lakes with multiple nests of malignant cells. Due to treatment with paclitaxel and carboplatin before surgery, the cellular component is relatively low (<5%).

**Fig. 6:**
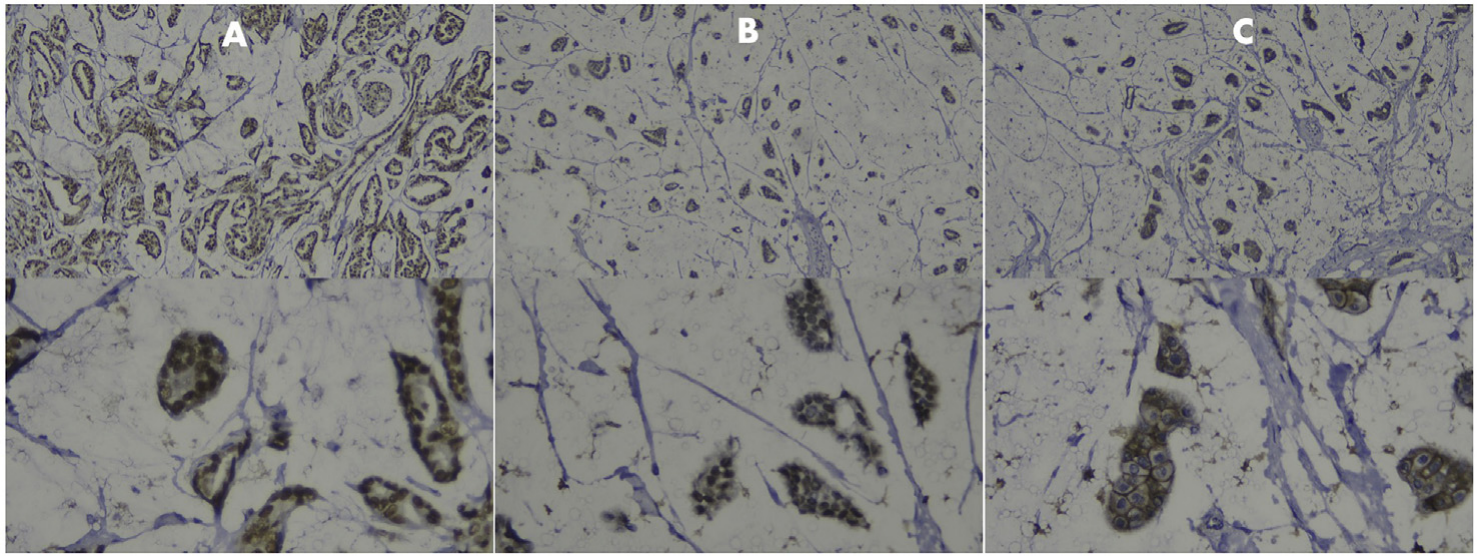
Immunohistochemistry staining panels showed ER expression was positive in the 90% of neoplastic cells (A), PR was positive in the 90% of the malignant cells (B), and Her-2 was positive in the 50% of the malignant cells (C).

A 46-year-old Javanese female presented in the emergency room with shortness of breath. Since the past month, the dyspnea got worse with physical activity and sleeping in supine or left lateral recumbent position and alleviated with sitting or left right recumbent sleeping position. Full examination of airway, breathing, and circulation revealed pleural effusion in the right hemithorax (Fig. 1) and a lump of 8 × 7x3 cm in size with skin ulceration and retracted nipple in the right breast as well as enlargement of right axillary lymph nodes. More detail history taking revealed that the patient recognized a lump around 2 cm in diameter since a year before admission. The lump grew slowly, and skin ulceration appeared since the past month. No previous history of gynecological cancers as well as benign tumor in the breast including fibrocystic, ductal papiloma and atypical ductal hyperplasia. Her personal history included G3P3, menarche at 14 years, breast feeding for 18–24 months of each child, and using of hormonal contraception for 4 years. No family history of breast and gynecological cancers as well as no other important former medical visits were reported. Pleural aspiration was performed resulting in 200 ml serous-hemorrhagic fluid. Cytology analysis of pleural fluid revealed ductal cell carcinoma. Fine-needle aspiration of the lump near the ulceration in the right breast also showed ductal cell carcinoma. Ultrasonography of the breasts revealed diffuse spiculated masses in the right breast with enlargement of multiple lymph nodes in the right axillae (Fig. 2). Mammogram of the right breast could not be performed because of the skin ulceration and mammogram of the left breast showed breast dense of D type with equal density lesion in the retro-papillae suggested a fibrocystic lesion (Fig. 3). The patient was then diagnosed as breast carcinoma T4bN1M1. Metastatic breast cancer was established from the finding of breathless accompanied by pleural effusion in the right hemithorax with cytology examination revealing positive ductal carcinoma, lump with skin ulceration in the right breast with fine needle aspiration biopsy confirming positive ductal carcinoma. The patient was then treated with 6 cycles of paclitaxel (175 mg/m^2^) and carboplatin (300 mg/m^2^). Partial response to the chemotherapy was observed shown by resolution of skin ulceration without significant reduction of tumor size. Modified radical mastectomy (with preserving pectoral, serratus, and intercostal muscles) and axillary node level I-II clearance were performed because of the clinically positive axillary nodes after chemotherapy (Fig. 4). Tumor histology revealed mixed colloid breast carcinoma as shown in Fig. 5. Pathology examination revealed a prominent white-brown soft-springy mass (7 × 5x2 cm in size) within a breast tissue with silk yarn marking in the axillary tail. Microscopically, ductal carcinoma arranged in tubular, acinar, and papillary patterns showing infiltration to the surrounding tissues (adipose, vascular, and lymphatic systems) within some large pools of mucins. Of the 11 axillary lymph nodes, 5 were positive of malignant cell infiltration containing mucinous deposition. Pathological section in the nipple areolar complex showed cancer cell infiltration in the dermis. The surgical margin was clear with a distant of more than 1 mm. In addition to the large deposition of mucin, tumor cells were rated as scant with cellularity <5% and significant fibrovascular components. Immunohistochemistry showed strong expression of ER (90% positive staining in the cancer nuclear cell), PR (80% positive staining in the cancer nuclear cell), and Her-2 (completely intense staining of cellular membrane in 50% of cancer cells thus was considered as positive 3+) (Fig. 6). Twenty-five cycles of adjuvant radiotherapy were delivered to reach a total dose of 50 Gy. Hormonal therapy with Tamoxifen 20 mg/day was then scheduled to the patient. The hormonal therapy was maintained and planned for 5 years. Surveillance was performed by regular monthly visit for physical examination. Chest X-ray, sonography for the breast and abdomen, liver function test, alkaline phosphatase, and blood based biomarker of Ca-153 was performed every 6 month. Bone survey was performed every year and biannual contralateral mammography. In 2 years after initial diagnosis, patient was performing well in daily life activity and was also returning to work without any sign of locoregional recurrent disease as confirmed by sonography of the breast and mammogram of the left breast. Abdominal sonography and bone survey showed no sign of liver and bone metastases. Chest X-ray showed residual pleural effusion in the latero-basal aspect of the right hemithorax. However, the blood-based biomarker Ca-153 remained high (201.8 U/mL after chemotherapy-surgery and 172.3 U/mL at 2 year follow up, with reference value < 25 U/mL).

## 3 Discussion

Colloid carcinoma is a rare variant of invasive breast cancer that represents only around 2% of total malignant breast tumor [2,4]. According to the mucin content, colloid breast carcinoma was grouped into pure if the mucin content comprises more than 90% and mixed colloid cancer if the mucin content is around 75–90% although the clear cut-off is still in debate [1]. Colloid breast carcinoma is considered as slow-growing tumor and usually has better prognosis [2,3] although clinicopathological variables are not significantly different with the typical invasive ductal carcinoma [2,6]. Local recurrence and distant spread are rarely found in colloid breast carcinoma and occur only in around 6% of total cases [1,2]. We reported a rare case of colloid breast carcinoma that was initially diagnosed as metastatic breast cancer in the pleura. Two previous studies have described the significant risk factor for local and distant spread of colloid breast carcinoma which is axillary lymph node involvement [1,7]. Larger tumor size is not associated with risk factor for distant spread because of large deposition of mucin in colloid breast carcinoma [1,2]. Further studies suggested that Her-2 [8], Ki-67, Topo-II, and p53 expression levels were associated with more aggressive behavior in colloid breast carcinoma [2].

Mammograms in colloid breast carcinomas typically show oval or lobular mass with circumscribed or microlobulated margin without presence of microcalcifications [9]. Breast sonography in colloid breast carcinoma shows typical features of isoechoic masses in pure type and hypoechoid masses in mixed colloid breast carcinoma [9]. In our case, mammography was not eligible because of large skin ulceration in the affected breast. Sonography revealed hipo-echoid diffuse mass with spiculated border and enlargement of axillary lymph nodes (Fig. 2). Systemic chemotherapy with carboplatin and paclitaxel was chosen due to the presence of pleural metastasis. Resolution of skin ulceration was not accompanied by significant reduction of tumor size and axillary lymph nodes after chemotherapy. Because colloid breast carcinoma shows low frequency of nodal involvement, slowly growing cancer, and relatively superior prognosis, some studies have suggested to avoid axillary clearance even in locally advanced cancer [10,11]. In addition, colloid breast carcinoma is usually found in elderly patients [11] with low rates of relapse after a long-term follow-up [2]. Therefore, radical surgery and aggressive chemotherapy are not always required [10,11]. However, with a potential aggressive behavior in younger patient with Her-2 overexpression as shown in our case, palliative surgery with mastectomy and axillary node dissection were an option because previous systemic therapy showed complete resolution of the pleural metastasis. Although axillary clearance might not improve survival, it removes the axillary disease and prevents locoregional recurrence [12,13]. In addition, colloid breast carcinomas frequently show resistance to radiotherapy and Her-2 inhibitors [14,15]. Palliative surgery is frequently selected for locally advanced and metastatic colloid breast carcinoma to improve patient's wellbeing and quality of life [10] although the clinical benefits to prolong overall survival is still unconfirmed [16]. The relative advantages of surgery in metastatic breast cancer have been established mostly from retrospective studies [16]. Therefore, the decision for surgery in metastatic breast cancer should be individualized for selected patients in relatively young women without presence of comorbidity.

Tumor histology of mastectomy after chemotherapy showed large pool of mucins with low cellular components (<5%) (Fig. 5). In accordance with our result, Jang et al. [17] and Didonato et al. [18] showed that tumor size was not significantly reduced after chemotherapy due to large deposition of mucins that did not dissolve upon systemic treatment. However, histology showed decreased cellularity components upon chemotherapy indicating pathological response [18].

After mastectomy and radiotherapy, hormonal therapy was delivered to the patient. Local control and complete resolution of pleural metastasis was still observed after 1-year follow-up. In accordance to the existence literature [14], the patient showed good response to hormonal therapy. Although the Her-2 expression was positive, we did not allocate trastuzumab to our patient because of previous report revealing resistance [14,15] although another report showed an excellent response to trastuzumab in colloid breast carcinomas with signet ring cell differentiation [17]. Several mechanisms underlying trastuzumab resistance are associated with mucin production as *MUC4* plays as extra-epithelial mechanical barriers to the antibody binding. In addition, *MUC4* is able to directly bind to Her-2 receptor causing alteration of the cellular signaling [19]. Her-2 expression in colloid breast carcinomas therefore indicates more aggressive behavior with higher probability for resistance to trastuzumab.

In our case, the patient complained of a lump at the right breast one year before she decided to seek medical help in the emergency room because of sort of breath and skin ulceration at the right breast. The significant delayed time of diagnosis might also contribute in the progression of colloid breast carcinomas. Earlier studies show the consequences of delayed diagnosis on breast cancer prognosis in which increased time delay is associated with more advance stage at diagnosis and poor survival [20,21]. Two major causes of diagnosis delay in breast cancer are patient factors which are related to misconception of cancer, low cancer awareness, use of traditional therapy, fear of treatment, and financial limitation as well as health system factors which are associated with limited health facilities, complicated referral health system, and appointment delay [21]. This case might also implicate the importance of public health actions to improve cancer awareness [22] and to take pragmatic steps to reduce delay diagnosis of breast cancer in Indonesia.

## Ethics approval

Not applicable.

## Sources of funding

SLA received PPUPT (2274/2019) grant from the Ministry of Research and Technology Republic of Indonesia, Dana Masyarakat (1499/2019) grant from UGM, and NUS-UGM-Tahir Foundation seed grant.

## Authors’ contributions

SLA conceptualized the report and finalized the manuscript. EKD provided and gave expertise in the tumor histopathology. WSA and LC gave expertise in the imaging. SLA, S, and TA were involved in the surgery and care of the patient. All authors read and approved the final manuscript.

## Registration of research study

Not applicable.

## Guarantor

SLA.

## Provenance and peer review

Not commissioned, externally peer reviewed.

## Availability of data and materials

The clinical and imaging data supporting the analysis and findings of this study will be available from the corresponding author upon reasonable request.

## Consent for publication

Written informed consent for reporting the case and any accompanying images was acquired from the patient. A copy of the written informed consent is available for review by the Editor-in-Chief of this journal. Patient identifying related material was not used in this manuscript.

## Declaration of competing interest

All authors have declared for no existence of any potential competing interest.
